# Association of Individual and Contextual Factors for Protection from Dental Caries

**DOI:** 10.3390/ijerph23030379

**Published:** 2026-03-17

**Authors:** Lívia Guimarães Zina, Priscila Almeida Rodrigues, Danúbia Aparecida de Miranda Matos, Luíza Moreira Silva, Rosana Leal do Prado, Rafaela Silveira Pinto, Janice Simpson de Paula

**Affiliations:** 1 Faculty of Dentistry, Universidade Federal de Minas Gerais (UFMG), Belo Horizonte 31270-901, Minas Gerais State, Brazil; luizamoresi12@gmail.com (L.M.S.); rosanahb@gmail.com (R.L.d.P.); rafaelasilveirapinto@gmail.com (R.S.P.); janicesimpson@ufmg.br (J.S.d.P.); 2 Municipal Government of Belo Horizonte (Brazil), Faculty of Dentistry, Universidade Federal de Minas Gerais (UFMG), Belo Horizonte 31270-901, Minas Gerais State, Brazil; priarod@gmail.com; 3 Municipal Government of Paraopeba (Brazil), Faculty of Dentistry, Universidade Federal de Minas Gerais (UFMG), Belo Horizonte 31270-901, Minas Gerais State, Brazil; danubiamatos28@hotmail.com

**Keywords:** dental caries, protective factors, child, dental health surveys, health promotion

## Abstract

**Highlights:**

**Public health relevance—How does this work relate to a public health issue?**

- Early childhood dental caries remains a common and socially patterned public health problem.
- This study analyses protection from caries using a salutogenic, health-promotion framework.

**Public health significance—Why is this work of significance to public health?**

- Individual socioeconomic factors were more strongly associated with caries-free status than contextual municipal factors.
- The findings reinforce equity as a central principle in childhood oral health promotion.

**Public health implications—What are the key implications or messages for practitioners, policy makers and/or researchers in public health?**

- Policies should prioritize reducing individual-level social inequalities to increase the number of caries-free children.
- Salutogenic approaches may strengthen oral health strategies beyond traditional risk-focused models.

**Abstract:**

Background/Objectives: Dental caries remains a global challenge, with high prevalence among five-year-old children and regional inequalities. The aim of this study is to identify factors associated with protection from dental caries in five-year-old children, using Salutogenic Theory as a reference. Methods: Secondary data were analyzed from the Minas Gerais Oral Health Survey. Five-year-old children (*n* = 1193) were examined. Parents or guardians answered a questionnaire addressing individual variables and the use of dental services. Dependent variables were the absence of caries activity (ACA) and absence of caries experience (ACE), which were extracted from the decayed–missing–filled primary teeth (dmft) index. Logistic regression analysis was used to estimate odds ratios with 95% confidence intervals for each block of variables on the hierarchical levels. The Complex Samples module of the SPSS 19.0 program was used. Results: Five hundred ninety-five children (50.5%) in the overall sample were caries-free. In the final model, white skin color, monthly family income greater than R$1500, having more than six material goods, and not visiting a dentist in the previous year were associated with ACA and ACE. Conclusions: Factors related to socioeconomic conditions were associated with protection from dental caries in early childhood, supporting equity-based public policies to increase the number of children not affected by caries.

## 1. Introduction

Although avoidable, dental caries continues to be one of the most common childhood diseases [1]. When untreated, caries can cause pain and suffering as well as compromise oral functions, affecting the quality of life of children [2]. While an accentuated decline has been seen in the occurrence of dental caries throughout the world in recent decades, studies have demonstrated that the prevalence remains high among five-year-old children, with even a slight increase in some countries [3,4,5,6]. Assessing dental caries at this age is important because it corresponds to the index age for primary dentition recommended by the World Health Organization and reflects early exposure to biological and contextual determinants, serving as a predictor of health inequalities and guiding public health actions [7].

A comprehensive review involving studies from Asia, Europe, South America, Africa, the Middle East, and Oceania revealed divergences in the prevalence of dental caries at five years of age, ranging from 22.5% (India) to 90% (Indonesia), with approximately two-thirds of the studies reporting caries in more than 50% of five-year-old children [1]. This condition still affects more than half of the child population in countries of Latin America and the Caribbean in the 21st century [5].

Access to oral health services in the Brazilian public health system is constitutionally guaranteed to the entire population, including routine preventive and curative oral health appointments for children of all ages; however, it faces several barriers, such as geographical inequalities, insufficient funding, and suboptimal private sector–public sector collaboration [8]. The most recent epidemiological oral health survey conducted in Brazil in 2023 showed that only 53.2% of five-year-old children were free of caries [3], with the highest values in the Southeast and South regions, and no improvements were observed when compared with the 2010 survey [9]. According to data from the 2012 Minas Gerais Oral Health epidemiological survey, similar results are found in the state of Minas Gerais as those reported for Brazil as a whole and the southeast region, with 50.5% of five-year-old children caries-free [10]. However, inequalities occur in caries experience at this age in the different regions of the state [10] and country [3].

The heterogeneity in the distribution of caries in the world, as well as in different regions of Brazil and of Minas Gerais state, demonstrates that caries cannot be explained by an individual biological focus alone; it is necessary to understand socioenvironmental determinants in the causal chain of the disease [11,12,13].

Considering the dental caries conceptual model in children [14], it is important to understand childhood health as the result of the interrelations of several factors related to quality of life, such as adequate dietary and nutritional status, oral hygiene behavior, parents’ employment and schooling, housing and sanitation, a healthy physical environment, and social support from the family [4,15,16]. These factors can be positive or negative and can determine or influence individual behaviors and are therefore denominated “risk” or “protection factors”, depending on their effect [17].

To gain a better understanding of the dynamics of the health-disease process, it is fundamental to have knowledge of factors that are favorable to the maintenance of results that are beneficial to health, even in the occurrence of adversities. Salutogenesis Theory (saluto = health; genesis = origin), which was developed by Aaron Antonovsky [18], states that health promotion factors are different from those that modify the risk for specific diseases [19,20]. According to the original idea, concentrating on resources and the capacity to generate health is more important than focusing on the causes of disease [21].

Gaps in the literature remain regarding the salutogenic approach to the prevention of dental caries. It is pertinent to reflect on the socioenvironmental, economic, and family contexts of five-year-old children who are free of caries to identify possible protection factors that can increase the possibility of positive outcomes and diminish the negative consequences of exposure to risk [12,22,23,24]. Such knowledge is important for guiding strategies for the strengthening of individuals, the definition of priorities, as well as the planning and implementation of actions that favor health, such as the integration of means of assessing oral health and family health in individuals [19,23]. All knowledge and its possible effect regarding modifiable factors on both the contextual and individual levels ultimately lead to healthy environments and the empowerment of the population.

Therefore, the aim of the present study is to investigate individual and contextual factors associated with protection from dental caries in five-year-old children using Salutogenic Theory as a reference.

## 2. Materials and Methods

A population-based, observational, cross-sectional study was conducted using secondary data from the Minas Gerais Oral Health Survey, which was an epidemiological survey to obtain knowledge on the prevalence of the main oral health problems of the state population and related factors. The method of the survey followed the guidelines employed in the Brazilian National Oral Health Survey [9] and internationally standardized by the World Health Organization [7]. Further details can be found in the survey report [10] and published methodological article [25].

The Minas Gerais Oral Health Survey was conducted in 2012 and included a sample of 1193 five-year-old children, with representativeness on the state level in three different domains: the state capital and two groups of municipalities denominated Interior I and Interior II [10]. For the capital, the data were extracted from the 2010 National Oral Health Survey conducted two years earlier. For the 60 municipalities from Interior I and II, the representative sample was based on the mean number of decayed, missing, and filled primary teeth (dmft) according to data from the 2010 National Oral Health Survey for the southeast region of the country [10].

The prevalence of dental caries in the five-year-old children was determined using the decayed–missing–filled teeth (dmft) index for the primary dentition. Oral examinations were performed by examiners who had undergone training and calibration exercises in the use of the diagnostic criteria proposed by the World Health Organization [7]. Inter- and intra-examiner agreement values were greater than 0.65 [10].

For the present investigation, the dependent variable was the absence of dental caries, which was assessed using two outcomes: absence of caries activity (ACA) (component “d” of the index = 0) and absence of caries experience (ACE) (components “d”, “m”, and “f” of the index = 0).

Contextual variables available in public databases were investigated considering Salutogenic Theory and scientific evidence on factors that can exert an impact on dental caries throughout the lifecycle of children [3,11,12,22,23,24].

The independent variables on the individual level (sociodemographic characteristics and the utilization of oral health services) obtained through the administration of questionnaires to parents or guardians in the Minas Gerais Oral Health Survey were sex (female or male), skin color (white or non-white), income (> or ≤R$1500 [Brazilian currency]), number of material goods (≥ or <6), crowding (≤ or >1.64 residents per room in the home), visit to the dentist (yes or no), reason for visit (prevention or treatment), frequency (<1 or ≥1 year), type of service (public or private/other) and evaluation of service (positive or negative). Material goods refer to household durable consumer items owned by the family, used as an indirect indicator of socioeconomic status. In the SB Minas survey, this variable was measured through the number of goods present in the household, including items such as televisions, refrigerators, sound systems, microwave ovens, washing machines, cars, and portable electronic devices (e.g., laptops and mobile phones). The total number of these items is used to characterize household living conditions and economic resources. The categorization of these variables considered the greater change in the absence of dental caries, as employed in the literature [3,26].

**Figure 1:**
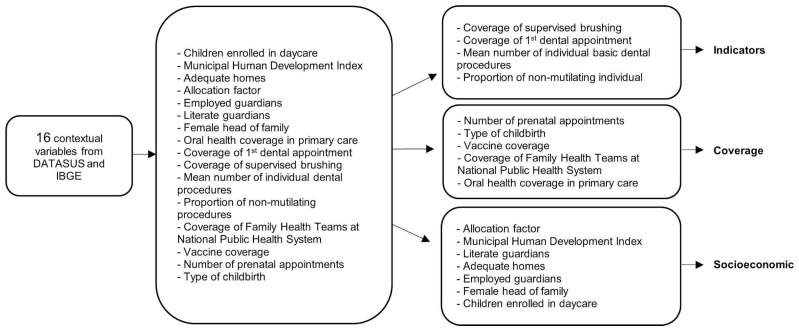
Grouping of variables through principal component analysis—PCA.

Characteristics related to the municipalities of the state of Minas Gerais in 2012 were extracted from public databases made available by the Informatics Department of the public healthcare system (DATASUS) [27] and the 2010 Brazilian census [28]. Sixteen variables were collected on oral health services, general health services, and socioeconomic indicators. Due to the large number of contextual variables, Principal Component Analysis (PCA) and Varimax rotation were performed [29], pooling the variables into three groups (Figure 1).

The null model of the multilevel analysis revealed that the contextual variables were not associated with the outcome, which impeded the use of multilevel analysis [30]. To assess the presence of contextual variability, a null mixed-effects Poisson model was initially estimated, considering municipalities as the clustering unit. The variance of the random intercept was very small for both outcomes (absence of caries activity and absence of caries experience). Furthermore, the likelihood ratio test comparing the mixed-effects model with the single-level Poisson model was not statistically significant (*p* = 0.159 for absence of caries activity and *p* = 1.000 for absence of caries experience). These results indicate little variability among municipalities and suggest that the use of a multilevel model would not substantially improve the model fit. Thus, a hierarchical logistic regression model was used, considering only variables on the individual level. Four initially chosen variables were not incorporated in the hierarchical model due to a response rate of around 50%, rendering the analysis invalid (reason for visit to dentist, frequency, type of service, and evaluation of service). A model for the determination of the absence of dental caries was run using the conceptual hierarchical modeling proposed by Victora and collaborators [31], which considered the existence of proximal and distal factors associated with the outcome. The proposed model considers distal factors to exert an influence on proximal factors, measuring their effect and controlling for possible confounding factors.

**Figure 2:**
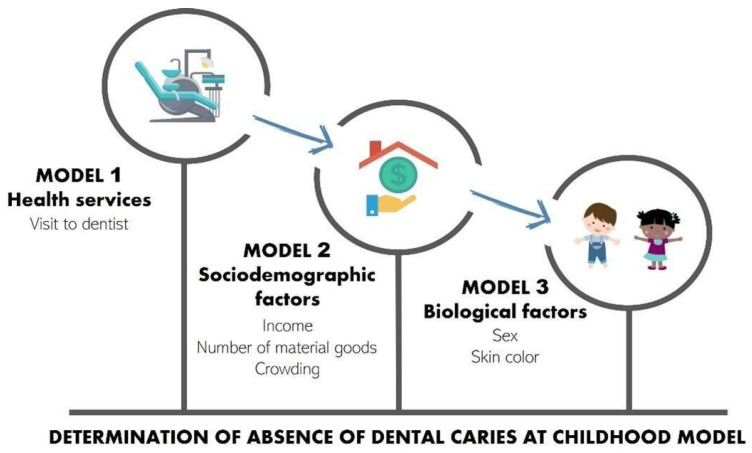
Hierarchical analysis model for the determination of the absence of childhood dental caries.

Model 1 included the variable on the distal level (utilization of oral health services). Model 2 also included variables on the intermediate level (sociodemographic characteristics). Model 3 also included variables on the proximal level (biological factors). The theoretical model guided the composition of the blocks of variables and their input order (Figure 2). Bivariate analysis was performed between the individual variables and outcome, and those with a *p*-value > 0.20 in this step were incorporated into the hierarchical analysis. Logistic regression was then performed in the distal block. Variables in this block with a *p*-value > 0.20 were incorporated into the analysis of the proximal hierarchical level. The same procedure was performed for all blocks. Unadjusted and adjusted odds ratios (OR) were estimated with respective 95% confidence intervals (CI). All analyses were performed with the aid of SPSS Statistics (IBM, Armonk, NY, USA), version 19.0.

This study is based on secondary data from public databases and, therefore, did not require approval from an institutional review board. The individual data were obtained from the public databank of the Minas Gerais Oral Health Survey, which had received approval from the institutional review board of Pontifícia Universidade Católica de Minas Gerais (process no. 9173, date of approval: 28 March 2012; certificate of approval: 01107412.4.0000.5137).

## 3. Results

Table 1 displays the results of the descriptive and bivariate analyses of the three blocks of variables (health services, sociodemographic characteristics, and biological factors) associated with the absence of caries activity (ACA) and the absence of caries experience (ACE).

The results of the hierarchical analysis of the ACA outcome are presented in Table 2. Children with white skin color, a higher family income, and a greater number of material goods, as well as those who had not visited a dentist in the previous year, were more likely to have an absence of caries activity. Similar results were found in the hierarchical analysis for the ACE outcome (Table 3).

## 4. Discussion

The present study identified factors associated with the absence of dental caries and therefore protectors from the disease using representative population-based data from the second most populous state in Brazil. Variables on the individual and contextual levels were investigated using the salutogenic approach and lifecycle theory. The analyses revealed no associations between the contextual variables (representative of the municipalities) and outcomes, suggesting that such variables are not associated with the prevention of dental caries.

In Brazil, dental health care for children is primarily provided through the national public health system, the Unified Health System (SUS), which guarantees universal and free access to health services. Oral health care is delivered mainly through Primary Health Care, particularly within the Family Health Strategy, where Oral Health Teams provide preventive and curative services, including dental examinations, health education, fluoride application, and restorative treatments [8]. In addition, school-based programs and community initiatives aim to promote oral health and prevent dental caries among children. Despite these efforts, inequalities in access to dental services and variations in service availability across regions still represent important challenges for the effective delivery of oral health care to the pediatric population [3].

The present results are in agreement with data in the literature demonstrating that context is not as much of a determinant of caries as individual socioeconomic factors. While some studies have reported the influence of contextual factors on oral health [32,33,34,35,36], these factors seem not to be addressed in such a way as to strengthen possible protective factors against the occurrence of dental caries in children [37,38].

In the present study, children identified as having white skin color were more likely to present an absence of active dental caries and no experience of caries. This is in agreement with findings described in previous studies conducted in Brazil as well as in other countries [39,40,41], which found a greater proportion of white children free of caries. The association between skin color and indicators of caries is complex and should be interpreted within the context of race/skin color as a social construct, reflecting historical and structural inequities in Brazilian society [42,43]. Evidence suggests that these differences are largely explained by disparities in socioeconomic conditions and access to goods and services among racialized groups, rather than any biological mechanism [3,44,45].

Another important aspect is that the measurement of ethnicity is quite fluid in studies that assess health disparities, as skin color may be self-declared or determined by an observer. Self-declarations are normally based on predetermined categories and reproduce personal associations with shared cultures and modes of living [46,47]. In contrast, skin color/ethnicity attributed by an observer may better capture differences in how individuals are socially perceived and treated, which may reflect processes related to discrimination and unequal access to social and health resources [48,49]. In the 2012 Minas Gerais Oral Health Survey, skin color was self-declared using the criteria defined by the Brazilian Institute of Geography and Statistics, which may represent cultural behaviors aggregated to the racial group. In this context, race/skin color should be interpreted as a proxy for social and structural disadvantage rather than a biological determinant of oral health outcomes [50].

Regarding sociodemographic characteristics, a higher family income, greater number of material goods, and less household crowding were predictors of a good oral health status in the children. The association between income and the distribution of dental caries is well established in the literature [12,51,52,53,54,55,56]. A higher income may be associated with a higher level of parents’ schooling, greater value placed on health and lifestyle, and greater access to health-related information, which may be an indirect factor in the determination of caries [52]. However, the literature is not always consistent regarding these relationships. A study conducted in Sudan found a higher prevalence of dental caries among private school attendees and children from higher socioeconomic backgrounds [57]. It is important to note that variations in caries prevalence may partly reflect differences in dietary habits, salivary microbiology, cultural practices, and oral hygiene behaviors across communities and are also associated with a range of socioeconomic and biological risk factors [58]. Likewise, a lower number of material goods and a higher number of residents in the home are proxies of unfavorable living conditions [33] and the level of family well-being in the home. From the standpoint of Salutogenesis Theory, access to resources and the capacity to use such resources are more important than prioritizing risk factors and behavioral changes [19]. According to the central concept of the model (sense of coherence), individuals who have a greater capacity to cope with stressors in life have a better chance of maintaining their own health [21]. Thus, psychological and social factors, such as family cohesion, religiousness, and resilience, are more determinant than contextual factors [14,23,56,59,60,61].

Not having been to a dentist in the previous year was significantly associated with the absence of dental caries in the children. This finding may be explained by the perception of parents regarding the oral health of their children, as many fail to take their children for preventive care in the absence of pain symptoms or the observation of caries [55,62,63]. A study conducted in the state of North Carolina in the United States found a significant association between age at the first dental appointment and the absence of caries, as children who first visited a dentist at 37 to 60 months of age had a significantly lower burden of the disease in comparison to those who visited a dentist prior to 24 months of age [64]. It is possible that children without caries do not go to the dentist precisely because they do not have treatment needs.

Some studies have demonstrated a greater search for dental care in the occurrence of pain rather than for the purposes of prevention [33,65]. In the present study, it was not possible to evaluate the reason for seeking care, the frequency of seeking care, the type of service used, or the evaluation of the service due to the insufficient number of answers to such variables in the public database, which impedes a better understanding of the association between not going to the dentist and the absence of caries.

However, it is not clear in the literature whether differences in dental caries status are found in children who visit the dentist at earlier ages. Some studies have found no direct evidence that first care screening affects early caries experience in children [66,67]. Other studies, however, have shown that children with a history of missed appointments are at higher risk of dental caries, as they present more enamel disturbances, greater caries experience, and higher caries activity [68]. When considering a purely curative model of care, visiting the dentist may be associated with higher caries levels; however, this occurs due to the reactive nature of care and the way the disease is measured. Families in situations of greater social vulnerability tend to use dental services less frequently and are less likely to participate in educational activities. Additionally, groups with lower educational levels and income tend to have lower regular use of preventive health services and a greater reliance on emergency care [69,70]. Visiting the dentist per se does not reduce the incidence of caries if not accompanied by the promotion of good oral hygiene habits and controlled sugar intake [67].

Since cross-sectional studies do not enable the determination of a cause-and-effect relationship between access to health services and the prevention of disease, this indicator should be analyzed with caution. Prospective studies with a longitudinal design are needed for a better understanding of the influence of access to oral health services on caries prevention. Such studies should measure the impact of preventive dental care on the maintenance of oral health in children, which could guide the formulation of policies, services, and actions on the collective and individual levels. Nevertheless, this discussion does not invalidate the relevance of dental visits. It is important to note that the limited availability of dentists within the public health system may contribute to a greater burden of dental caries among children [71]. From a public health standpoint, maintaining the recommendation of early dental visits for children remains essential, together with the implementation of family-oriented oral health education strategies [16].

Most studies in the literature with secondary data evaluate individual and contextual factors from the standpoint of disease [13,72]. This is the first study involving the data bank of the Minas Gerais Oral Health Survey to evaluate the protection of oral health in childhood using the salutogenic approach. Most studies involving this approach analyze sense of coherence and locus of control [73,74] as predictors of protective health behaviors. However, oral health databanks do not usually contain such variables. The option was to find variables that could serve as adequate replacements through the paradigm of health promotion and its social determinants [39,75] accessible in public databanks along lines of the same health promotion rationale.

The strong points of the present investigation are the representativeness of the data as well as internal and external validity. Moreover, the analysis is robust and enabled the control for possible confounding factors. However, the main limitation regards the cross-sectional design, which enables the determination of associations between variables but does not permit the establishment of causality. In addition, the data were collected in 2010 and 2012. Nevertheless, these data remain relevant, as they originate from the only population-based oral health survey representative of the entire state of Minas Gerais, providing important evidence for understanding inequalities in oral health. Another limitation regards the absence of variables such as dietary habits and oral hygiene measures in the SB Minas database and the lack of sufficient information in the databases for some variables, which hindered a more global understanding of the effect of access to dental care on the prevention of caries.

The health promotion approach has led to significant results regarding the improvement in oral health status, beginning with the recognition that the development of dental caries is determined socially. Protective factors related to oral health are modifiable on the individual level but are also strongly influenced by sociopolitical factors that are beyond the control of many people. The salutogenic approach emphasizes the establishment of public policies that promote the oral health of individuals—those in early childhood in the present case—through population-based prevention strategies based on the recognition of protective factors for the disease in question [19]. With the premise that income, ethnicity, and access to goods are protective factors for dental caries, policies should promote the generation of income, expand the job market, improve sanitation and housing, and expand access to health services and education, consequently enabling the establishment of favorable conditions for the positive use of resources and contributing to a greater number of children not affected by the disease. Such policies should be based, above all, on equity on the individual level by exerting an influence on the social determinants of health and combatting disparities among children [76].

## 5. Conclusions

The findings support a salutogenic perspective by showing that protective factors related to favorable socioeconomic conditions are associated with the absence of dental caries in five-year-old children, highlighting the importance of public policies based on equity and strengthening social and environmental resources that promote oral health in early childhood.

## Figures and Tables

**Table 1:** Distribution of absolute and relative (%) frequencies in a sample of five-year-old children according to caries activity and caries experience for blocks of individual variables (biological, sociodemographic, and related to health services). Minas Gerais Oral Health Survey, 2012.

Variables			Caries Activity						Caries Experience					
			With No Active Caries (d = 0)		With Active Caries (d = 0)		Total	*p*-Value	No (dmft = 0)		Yes (dmft > 0)		Total	*p*-Value
			n	%	n	%			n	%	n	%		
Biological	Sex	Male	339	57.99 (52.83–62.99)	268	42.00 (37.00–47.16)	607	0.615	301	51.04 (45.91–56.16)	306	48.95 (43.83–54.08)	607	0.762
		Female	326	56.12 (50.12–61.94)	260	43.87 (38.05–49.87)	586		294	49.90 (44.20–55.60)	292	50.09 (44.39–55.79)	586	
	Skin color	Non-white	309	46.25 (40.96–51.63)	352	53.74 (48.36–59.03)	661	*p* < 0.001	278	40.86 (36.13–45.77)	383	59.13 (54.22–63.86)	661	*p* < 0.001
		White	356	70.37 (65.17–75.10)	176	29.62 (24.89–34.82)	532		317	62.32 (57.18–67.19)	215	37.67 (32.80–42.81)	532	
Sociodemographic	Crowding	>1.64	259	53.39 (47.08–59.61)	248	46.60 (40.38–52.91)	507	0.074	236	47.33 (41.82–52.91)	271	52.66 (47.08–58.17)	507	0.118
		≤1.64	406	60.21 (55.20–65.01)	280	39.78 (34.98–44.79)	686		359	53.18 (47.86–58.43)	327	46.81 (41.56–52.13)	686	
	Income	≤R$1500	420	50.30 (45.50–55.10)	426	49.69 (44.89–54.49)	846	*p* < 0.001	379	44.30 (40.14–48.55)	467	55.69 (51.44–59.85)	846	*p* < 0.001
		>R$1500	222	72.07 (65.24–78.01)	83	27.92 (21.98–34.75)	305		194	63.77 (56.47–70.49)	111	36.22 (29.50–43.52)	305	
	Goods	<6	217	45.27 (39.63–51.03)	255	54.72 (48.96–60.36)	472	*p* < 0.001	195	40.27 (35.19–45.57)	277	59.72 (54.42–64.80)	472	*p* < 0.001
		≥6	448	63.61 (58.51–68.42)	273	36.38 (31.57–41.48)	721		400	56.15 (51.16–61.01)	321	43.84 (38.98–48.83)	721	
Health services	Visit to the dentist	No	310	59.89 (54.24–65.29)	208	40.10 (34.70–45.75)	518	0.156	309	59.84 (54.18–65.25)	209	40.15 (34.74–45.81)	518	*p* < 0.001
		Yes	350	55.06 (49.95–60.06)	315	44.93 (39.93–50.04)	665		281	44.09 (39.21–49.10)	384	55.90 (50.89–60.78)	665	

**Table 2:** Hierarchical analysis of individual factors associated with absence of caries activity (d = 0) in five-year-old children. Minas Gerais Oral Health Survey, 2012.

Variables		Model 1 * OR (95% IC) *p*-Value	Model 2 ** OR (95% CI) *p*-Value	Model 3 *** OR (95% CI) *p*-Value
Skin color	Non-white			1
	White			2.45 (1.77–3.40) *p* < 0.001
Income	≤R$1500		1	1
	>R$1500		2.17 (1.48–3.18) *p* < 0.001	1.89 (1.30–2.76) 0.001
Goods	<6		1	1
	≥6		1.80 (1.32–2.47) *p* < 0.001	1.78 (1.29–2.45) 0.001
Crowding	>1.64		1	-
	<1.64		1.25 (0.88–1.75) 0.209	-
Visit to the dentist	No	1	1	1
	Yes	0.82 (0.62–1.08) 0.156	0.68 (0.50–0.92) 0.013	0.67 (0.50–0.90) 0.004

* Model 1—health services. ** Model 2—health services + sociodemographic characteristics. *** Model 3—health services + sociodemographic characteristics + biological factors.

**Table 3:** Hierarchical analysis of individual factors associated with absence of caries experience (dfmt = 0) in five-year-old children. Minas Gerais Oral Health Survey, 2012.

Variables		Model 1 OR (95% CI) *p*-Value	Model 2 OR (95% CI) *p*-Value	Model 3 OR (95% CI) *p*-Value
Skin color	Non-white			1
	White			2.23 (1.68–2.96) *p* < 0.001
Income	≤R$1500		1	1
	>R$1500		2.03 (1.39–2.96) *p* < 0.001	1.76 (1.22–2.52) *p* < 0.003
Goods	<6		1	1
	≥6		1.78 (1.33–2.39) *p* < 0.001	1.77 (1.31–2.38) *p* < 0.001
Crowding	>1.64		1	1
	<1.64		1.33 (0.97–1.82) 0.077	1.26 (0.92–1.72) 0.144
Visit to the dentist	No	1	1	1
	Yes	0.53 (0.39–0.71) *p* < 0.001	0.42 (0.30–0.58) *p* < 0.001	0.40 (0.29–0.56) *p* < 0.001

## Data Availability

Data available in a publicly accessible repository. Data were obtained from the State Department of Health of the Government of Minas Gerais, Brazil, and are available upon request at the e-mail address saudebucal@saude.mg.gov.br.

## Abbreviations

The following abbreviations are used in this manuscript:

ACA	Absence of caries activity
ACE	Absence of caries experience
dmft	Decayed–missing–filled teeth
DATASUS	Brazilian public healthcare system database
IBGE	Brazilian Institute of Geography and Statistics
PCA	Principal Component Analysis
OR	Odds ratio
CI	Confidence interval
